# Therapeutic evaluation of a patient with ruptured intracranial aneurysm without subarachnoid hemorrhage by CT imaging: a case report

**DOI:** 10.1186/s12883-018-1197-y

**Published:** 2018-11-29

**Authors:** Guangyong Chen, Meiling Xu, Long Ma, Yufei Gao, Chengyan He, Jinnan Zhang

**Affiliations:** China-Japan Union Hospital of Jilin University, Jilin, China

**Keywords:** Subarachnoid hemorrhage, Intracerebral hematoma, Ruptured aneurysm, Imaging examination

## Abstract

**Background:**

The majority of ruptured intracranial aneurysms are combined with subarachnoid hemorrhage, but patients with only intracerebral hematoma without any subarachnoid hemorrhage are extremely rare.

**Case presentation:**

The patient was hospitalized due to sudden dizziness combined with slurred speech. The patient showed considerable decreased physical activity without any nuchal rigidity. Head CT showed hematoma in the left temporal lobe, and the shape of hematoma was extremely irregular. MRI indicated the absence of any vascular malformations. The patient was diagnosed with middle cerebral artery bifurcation aneurysm in the left by head CTA. Intracranial aneurysm clip and removal of hematoma in the left temporal lobe were performed under general anesthesia. The patient did not show any significant neurological dysfunction after the surgery and was followed up for 4 months after discharge with GOS score of 5 points.

**Conclusions:**

Intracranial hematoma with irregular morphology around the lateral fissure of the brain should be considered critical in order to avoid misdiagnosis and any possibility of missed diagnosis of vascular lesions, so as to ensure an exact therapeutic strategy with good prognosis for the patients.

## Background

There are two manifestations of intracranial aneurysm rupture: ① The first is simple subarachnoid hemorrhage (SAH), accounting for 85%. ② The second is intraparenchymal/intracranial hematoma (IPH/ICH), accounting for 15%, which can be combined with subarachnoid hemorrhage or intraventricular hematoma (IVH). Some previous case reports have indicated few cases, which are manifested as IPH and IVH without any SAH [1]. However, there are few cases with ruptured aneurysms associated with IPH, especially when the hematoma occurs in the brain tissues surrounding the lateral fissure, which can be easily confused with hypertensive intracerebral hemorrhage. Because the mortality and disability rates of intracranial aneurysm rupture are significantly higher compared to hypertensive intracerebral hemorrhage, significant differences exists in the therapeutic strategies between them. Thus, prognosis of patients will be seriously affected in case of any misdiagnosis or missed diagnosis. Therefore, intracranial hematoma with irregular shape around the lateral fissure should be considered critical and needs strategic therapeutic evaluation. In this study, we reported a patient with ruptured intracranial aneurysm who had simple hematoma in the temporal lobe without any subarachnoid hemorrhage.

## Case presentation

**Fig. 1:**
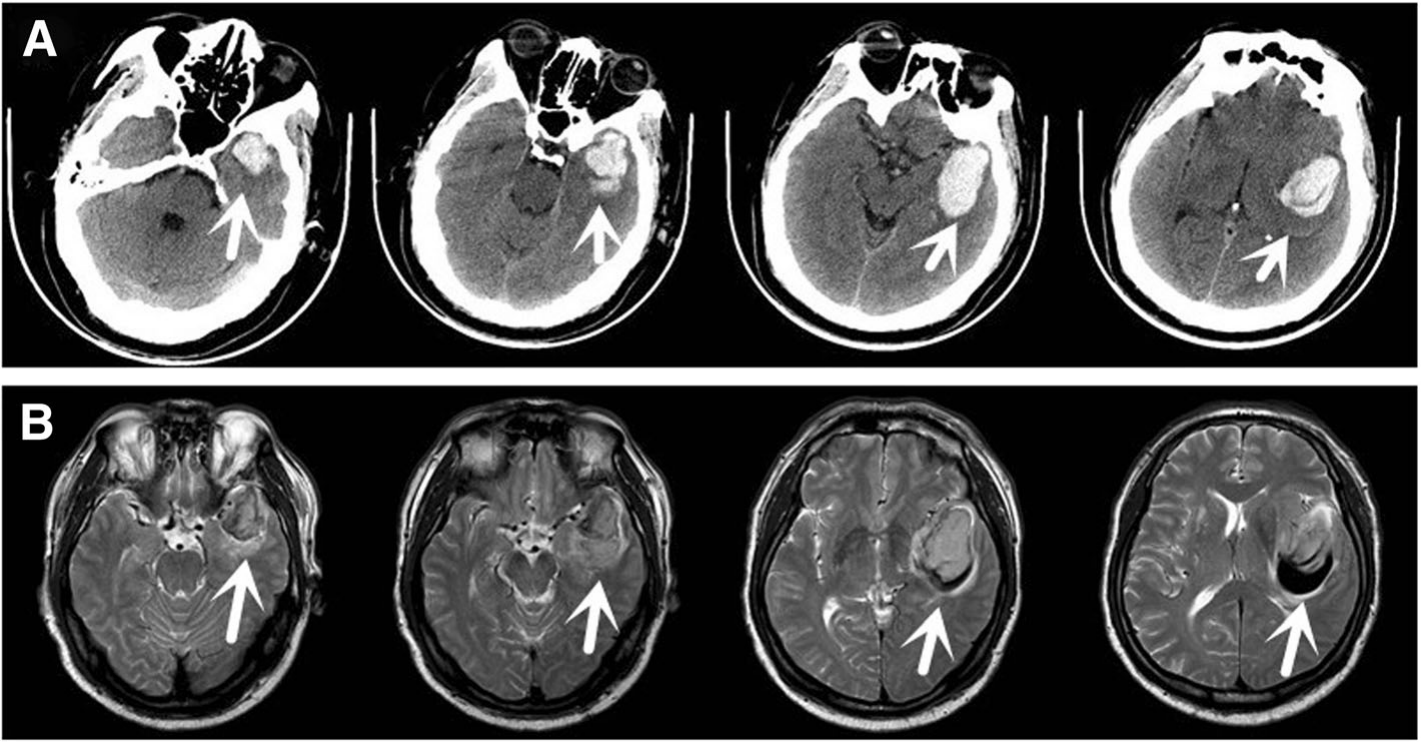
**a** Clump-like high-density shadows were visible in the left temporal lobe, and fissures of the left lateral ventricle and the left lateral fissure were narrowed due to compression. **b** Head MRI taken on the next day after onset showed that there was no significant vascular malformation

**Fig. 2:**
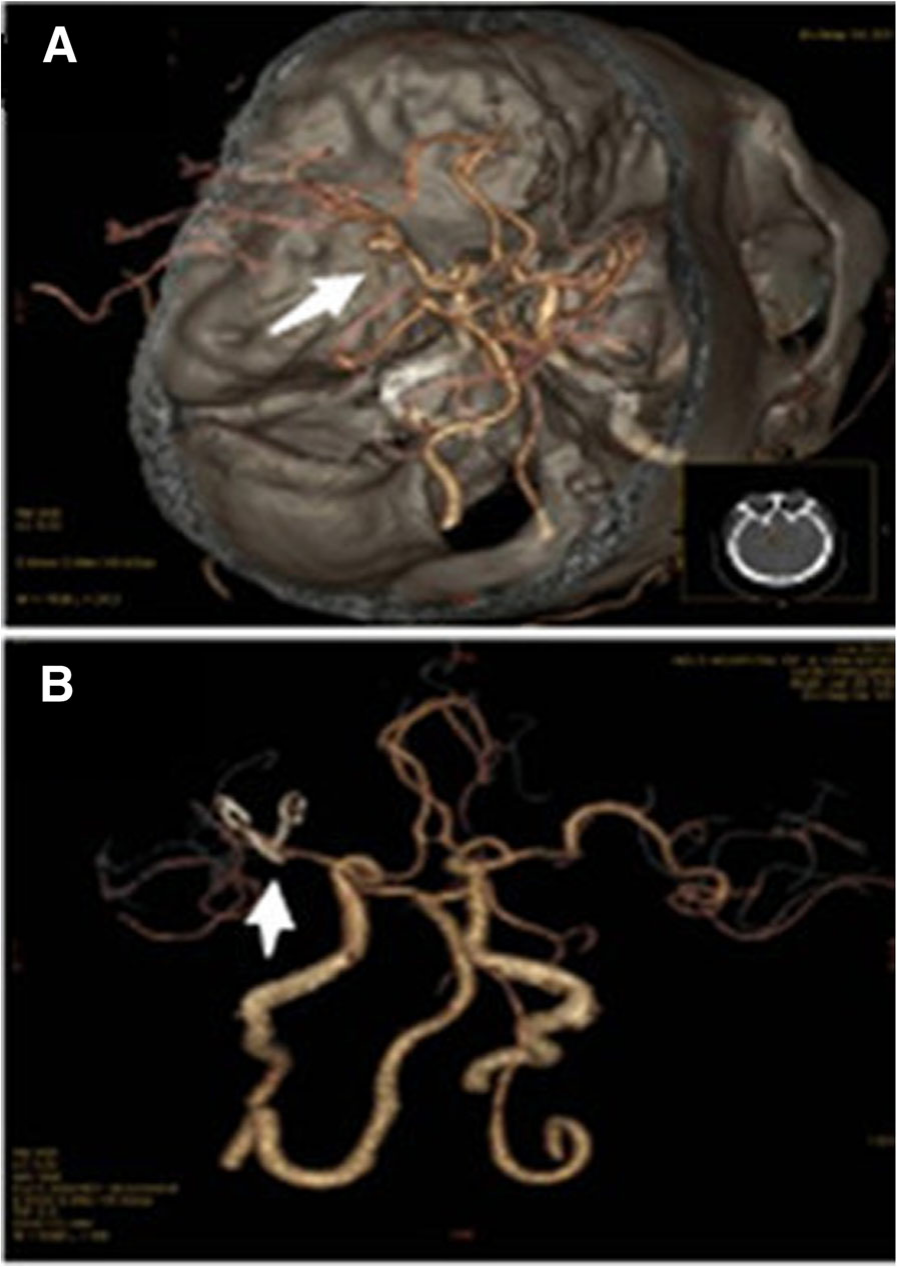
**a** CTA demonstrated that there was vesicle-like bulges in the left middle artery bifurcation and the formation of asci on the vesicle was visible. **b** Head CTA revealed that vessels in the middle cerebral artery bifurcation were in good shape, and no residual aneurysm neck and relapse were observed

The male patient aged 60 years was hospitalized due to sudden dizziness for 3 h combined with slurred speech on May, 16, 2017. The patient had nausea, but he did not have vomiting, incontinence and unconsciousness. The patient was immediately referred to China-Japan Union Hospital of Jilin University. Head CT performed within 3 h of onset of symptoms (Fig. 1a) showed hemorrhage in the left temporal lobe, and the patient was admitted in our department with a clinically confirmed diagnosis of “hemorrhage in the left temporal lobe”. The patient had a previous history of hypertension for more than 10 years, and was under medication to control and maintain the blood pressure to be around 130/80 mmHg. The patient denied the history of diabetes and had no bad habits, such as smoking and drinking alcohol. Physical examination at admission revealed that his body temperature was 36.2 °C and blood pressure was 196/119 mmHg. The patient was conscious and suffered from incomplete aphemia. Bilateral pupils were of the size and round, the diameter of pupils was 3 mm, and pupils were sensitive to light reflexes. Muscular strength of the limbs was about grade 4. Bilateral pathological signs were negative, and there was no significant abnormality noticed during nervous system examination. Head MRI conducted the next day after admission indicated no significant vascular malformations (Fig. 1b). General consultation evaluated that bleeding sites of the patient were slightly different from hypertensive intracerebral hemorrhage. The hematoma sites were close to the middle cerebral artery and the walking areas of its branches, mainly in the temporal lobes. Therefore, cerebral vascular hemorrhage and other causes could not be excluded, and head CTA was further suggested. Head CTA performed on 17th May 2017 showed that the patient suffered from intracerebral hematoma caused by aneurysm rupture at the M1 bifurcation of the left middle cerebral artery, without any significant subarachnoid hemorrhage (Fig. 2a). Craniotomy and clipping of intracranial aneurysm were performed the next day after admission. During the operation, the aneurysm was found to be located in M1 bifurcation of the left middle cerebral artery, which was in cystic bulging. An asci formation with the size of 3 × 4 × 5 mm was located on the upper side of the aneurysm, and the aneurysm neck measured 4.2 mm, which was clipped successfully. There was no significant hematoma in the subarachnoid space, and the arachnoid around the aneurysm showed no obvious adhesion during the surgery. The patient successfully recovered from anesthesia after the surgery, without any significant neurological deficits. The patient was discharged after 2 weeks and Glasgow Outcome Scale (GOS) scored 5 points. Follow-up was performed for 4 months at the Outpatient Department. Head CTA (Fig. 2b) demonstrated that aneurysm did not relapse and GOS score was 5 points.

## Discussion and conclusions

Most intracranial aneurysm rupture is usually reported to be associated with subarachnoid hemorrhage. There are few relevant case reports with manifestation of only IPH, IVH, SDH or IMH alone and without any SAH, which are mainly sporadic case reports [1]. It is not only a relatively difficult task to accurately diagnose aneurysm rupture in patients without SAH by performing a CT, but also is highly prone to misdiagnosis or missed diagnosis. Moreover, the diagnosis of these cases requires more clinical expertise. Because aneurysm rupture is usually combined with IPH or IVH and SDH, intracranial pressure of such patients is relatively higher compared to patients with simple SAH. Moreover, some patients may lose treatment opportunity due to deteriorated condition caused by sharp increase in intracranial pressure, and therefore, their prognosis may become much worse compared to patients with simple SAH [2, 3]. The patient in this study had hematoma in the left temporal lobe without SAH, which was confirmed by head CT scan at the time of admission. Moreover, the patient had a history of hypertension for many years, and the blood pressure at the time of admission was high, thus it was highly prone to be misdiagnosed as hypertensive intracerebral hemorrhage. Our medical team studied performances of head CT carefully, and found that hematoma was mainly located in the surrounding areas of the lateral fissure, adjacent to the left middle cerebral artery and its branches. Bleeding from intracranial vascular malformation or aneurysm rupture could not be excluded. Head MRI and CTA should be performed timely to avoid the occurrence of misdiagnosis and missed diagnosis, so as to ensure a good prognosis of the patient.

Ruptured aneurysms without SAH are relatively rare, and may arise due to several reasons. We believed that the causes can be broadly divided into two types, including ruptured aneurysms absolutely without SAH and ruptured aneurysms without SAH detected.

1. Aneurysms absolutely without SAH refer that the occurrence of aneurysm rupture is for the first time, and there is no traffic between aneurysm rupture and subarachnoid space. There are no blood cells, hemoglobin and hemosiderin in all stages of bleeding. Moreover, there is no imaging performance. Physical examination demonstrates that there is no sign of subarachnoid hemorrhage. This type of patients is very uncommon, and the possible cause may be due to the rupture of inner elastic membrane of the aneurysm and the expansion of the outer membrane. These changes may cause blood flow into vascular walls without breaking into the subarachnoid space, that may ultimately end up with giant mesenteric aneurysm. Another possibility reported include exudation of vascular protein through the vascular walls before blood flow breaks into the subarachnoid space. This may lead to aseptic adhesion of vasculature in the surrounding subarachnoid space, preventing the blood flow to break open into the subarachnoid space. Consequently, aneurysms rupture occurs as tumor-bearing artery wall thins constantly under the impact of blood flow [4–7]. Although the clinical case in our study had not reported any significant stiffness of neck by physical examination, the patient was not considered to belong to this type, because cerebrospinal fluid was mild red in color when subarachnoid space was examined during surgery.
2. Ruptured aneurysms without SAH detected indicates that aneurysm rupture occurs for the first time or multiple times, without any traffic between aneurysm rupture and subarachnoid space. In this case, the blood does not flow into the subarachnoid space due to various causes, or blood flows into the subarachnoid space without any favorable imaging manifestation and visible significant signs of subarachnoid hemorrhage detectable during the clinical examination. This clinical case study underlines considerable explanations to this unique neurological conditions with varying pathophysiology.

### 1.1 Time of CT scan

During the hyper-acute phase of hemorrhage (the initial 4-6 h), erythrocytes broken into the subarachnoid space are intact with oxy-hemoglobin and without any damage. During this phase, a CT scan cannot easily detect the presence of hemorrhage, because of a lack in contrast due to the same density with the brain tissue. In addition, the CT shows a continuous decrease in the sensitivity of SAH, and any further CT examination later after onset of symptoms, most likely lead to missed diagnosis of aneurysmal SAH [5]. In our case study, neither a head CT within 3 h after the onset of symptoms could detect SAH nor an MRI performed the next day could detect SAS, which suggest that there was no clinical correlation with the time of CT scan.

### 2.2 Amount of hemorrhage

If only a small amount of SAH is caused by aneurysm rupture, SAH would quickly fit into the subarachnoid space, thus SAH cannot be displayed by CT. In our study, preoperative head CT of this case showed the presence of hematoma in the temporal lobe, which was confirmed to be caused by aneurysm rupture happened during the surgery, thereby we concluded that undetectable SAH could not be correlated with the amount of hemorrhage.

### 2.3 The direction of aneurysm bleeding sites

The direction of rupture of the aneurysm towards the upper side may determine whether the bleeding occurs in the parenchyma, the ventricle or the cistern. If aneurysm rupture points to intracranial fissure with lower tension or larger space, blood would not flow into the subarachnoid space with relatively high tension after hemorrhage. In this case, after removing some hematoma during the surgery, examination of the middle cerebral artery and its branches showed that aneurysm ascus rupture was located in the facies medialis of the temporal lobe, pointing to the direction of hematoma in the temporal lobe. Therefore, undetectable SAH in this case was considered to be caused by the formation of aneurysm asci and the rupture pointing towards the temporal lobe.

### 2.4 Times of hemorrhage

Recurrence of hemorrhage may also be one among the various causes for aneurysm rupture without SAH [6]. The possible mechanism may be due to protein deposition occurring around the rupture of aneurysm after the initial bleeding form a fibrinous adhesion. In this study, intraoperative examination of the subarachnoid space surrounding the aneurysm and its asci did not detect adhesion, thus chances of any further recurrence of hemorrhage was excluded.

This case reminded us that, hematoma performance of head CT should be carefully examined to determine whether the hematoma sites were located in the walking areas of intracranial great vessels. Furthermore, the presence of intracranial aneurysms should be thoroughly evaluated to reduce the chances of misdiagnosis and missed diagnosis in order to ensure a good prognosis of the patients.

Nevertheless, due to the limitation of the number of cases in this study, conclusions derived from this single case study may only represent personal opinions of the author. Therefore, we suggest that clinical neurologists should carefully evaluate the necessity of various auxiliary examinations, so as to reduce unnecessary medical burden of the patients.

## Abbreviations

GOS: Glasgow Outcome Scale
IPH/ICH: Intraparenchymal/intracranial hematoma
IVH: Intraventricular hematoma
SAH: Subarachnoid hemorrhage
